# Epoxy Resin Composite Based on Functional Hybrid Fillers

**DOI:** 10.3390/ma7086064

**Published:** 2014-08-22

**Authors:** Mariusz Oleksy, Karolina Szwarc-Rzepka, Maciej Heneczkowski, Rafał Oliwa, Teofil Jesionowski

**Affiliations:** 1 Faculty of Chemistry, Rzeszow University of Technology, Al. Powstancow Warszawy 6, PL-35959 Rzeszow, Poland; E-Mails: mhen@prz.edu.pl (M.H.); oliwa@prz.edu.pl (R.O.); 2 Institute of Chemical Technology and Engineering, Faculty of Chemical Technology, Poznan University of Technology, M. Sklodowskiej-Curie 2, PL-60965 Poznan, Poland; E-Mails: karolinaszwarc1@wp.pl (K.S.-R.); teofil.jesionowski@put.poznan.pl (T.J.)

**Keywords:** epoxy resin, hybrid polymer composites, polyhedral oligomeric silsesquioxane (POSS), modified bentonite, modified silica, mechanical properties

## Abstract

A study was carried out involving the filling of epoxy resin (EP) with bentonites and silica modified with polyhedral oligomeric silsesquioxane (POSS). The method of homogenization and the type of filler affect the functional and canceling properties of the composites was determined. The filler content ranged from 1.5% to 4.5% by mass. The basic mechanical properties of the hybrid composites were found to improve, and, in particular, there was an increase in tensile strength by 44%, and in Charpy impact strength by 93%. The developed hybrid composites had characteristics typical of polymer nanocomposites modified by clays, with a fine plate morphology of brittle fractures observed by SEM, absence of a plate separation peak in Wide Angles X-ray Scattering (WAXS) curves, and an exfoliated structure observed by TEM.

## 1. Introduction

There have been many publications in recent years on the subject of hybrid polymer composites [1,2,3,4,5,6,7,8,9]. The main reason for the interest in this area is the possibility of developing materials with higher rigidity and impact resistance. Well-designed hybrid composites utilize the advantages of the individual components so as to minimize the defects arising from their separate use.

Undoubtedly, the continued development of hybrid composite materials, mainly nanocomposites, is associated with a search for better modifiers with unique functional properties, whose presence in the composite in small quantities significantly improves properties, such as fire resistance and thermal stability. In addition to well-known flame retardants, polyhedral oligomeric silsesquioxanes (POSS) are particularly noteworthy. Important features that make this an interesting research material include the presence of an inorganic silicon-oxygen core and organic functional groups located at the corners, and its small size of about 0.5 nm, where the size of the whole molecule with substituents amounts to 1–3 nm. Because of the cost of synthesis of octasilsesquioxanes, research is being carried out into their use as modifiers of conventional fillers, such as aluminosilicates or silica, which can then be used as functional hybrid nanofillers of polymers.

The main advantage of layered aluminosilicate (LAS) modified with octasilsesquioxane salts is its high thermal stability, due to the relatively high decomposition temperature of the modifier, which typically exceeds 300 °C. In recent years, many papers have been published [10,11] in which oligomeric silsesquioxane was used as a modifier of montmorillonites. They describe a method of preparation of polylactide nanocomposites containing montmorillonite (MMT) modified by protonated aminopropylisobutylsilsesquioxane (POSS-NH_3_^+^). Zhao* et al.* [12] used a quaternary ammonium salt of aminopropylisooctylsilsesquioxane to modify bentonite. The authors studied the effects of the type and amount of modifier on the degree of modification of bentonite, which was then used to prepare a composite based on polyamide 12. This case provided the greatest improvement in Young’s modulus (by about 60%), and a 10% increase in yield stress, compared with the unfilled polyamide 12. In turn, McLauchlin* et al.* [13] used a pair of surfactants in the form of aminopropylsilsesquioxane (AP-POSS) for modification of the bentonite Closite^®^ Na^+^. The resulting filler was used to produce a nanomatrix of poly(butylene terephthalate). In another study, Liu* et al.* [14] described a method of modifying montmorillonite using octaaminopropylsilsesquioxane. The modified filler was used to obtain epoxy nanocomposites, which displayed improved thermal stability. Fu* et al.* [15] used a process of polymerization in emulsion to obtain polystyrene nanocomposites, using three types of modified LAS, the modifiers being *N*,*N*-dimethyl octadecylamine, 4-vinylbenzyl chloride and trisilanolisobutylsilsesquioxane. The use of these modifiers led to the largest separation of aluminosilicate plates, from 1.29 nm to about 3.95 nm, and consequently the obtaining of composite polystyrene with the best functional properties. In another paper [16] the same authors performed a similar modification of bentonite. The resultant filler was used to obtain a polystyrene nanocomposite in which the glass transition temperature increased to 103–108 °C.

Silicas, like LAS, are very interesting fillers. They are used in many technological and industrial applications [17,18,19]. Due to its unique mechanical, thermal and dielectric properties, amorphous silica has become a key material used, e.g., in microelectronics [20], for the production of glass and ceramics, in nanotechnology and in the electro-chemical industry [20,21]. One of the most important properties of silicas is their significant sensitivity to chemical modifications. The modification of silica by organofunctional silanes has already been extensively tested and documented. A great deal of research has also been done on the chemistry of the formation of covalent bonds between the silanol groups of silica and a reactive group derived from organosilane or from a hydrolyzed group of a proadhesive compound [22,23,24,25]. Thus far, there are only limited reports in the literature on the use of POSS to functionalize the surface of silicas. For example, Carniato* et al.* [26] examined the immobilization of cellular titanosilsesquioxanes (Ti-POSS), both on the surface of ordered mesoporous silica (SBA-15) and on a silica with a disordered structure (SiO_2_-Dav). The aim of the study was to obtain active heterogeneous catalysts. The silica surface was modified with 3-isocyanatopropyltriethoxysilane (TSIPI) and a POSS compound containing reactive aminopropyl groups and ethoxy groups (Ti-NH_2_POSS). Bhagiyalakshmi* et al.* [27] presented an interesting example of bifunctionalization of the silica medium. On the surface of chlorofunctionalized mesoporous silica of type SBA-15 (Cl-SAB-15), octa(3-aminophenyl)octasilsesquioxanes (OAPS) were inoculated. As a result of their study, the authors concluded that this inoculation extends the range of possible applications of polyhedral oligomeric silsesquioxanes, and classified them as novel compounds used for the adsorption of carbon dioxide [27]. SiO_2_/POSS systems were also produced by Bianchini* et al.* [28]. Research was carried out to demonstrate the catalytic properties of metallocene silica modified by closed POSS structures in the process of ethylene polymerization. It was observed that the use of POSS-modified silica led to an increase in catalytic activity by about 50% compared with the unmodified medium. In addition, Szwarc-Rzepka* et al.* [29,30] have proposed not only the permanent connection of silicas with POSS compounds using organosilanes, but also direct modification of the silica surface by silsesquioxanes [31,32]. The resulting hybrid systems were used, for instance, as fillers in gel polymer electrolytes [33].

Our previous work on modified bentonite nanocomposites with a synthetic resin matrix [34,35,36,37] prompted us to undertake studies on the development of new epoxy composites with various fillers with different particle shapes. This provides a new way to combine POSS-modified bentonite (platelet shape) and POSS-modified silica (spherical shape), leading to functional hybrid composites. This was the aim of our recent patent notification [38]. The objective of the present work is to investigate the impact of the new synthesized nanofillers—POSS-modified bentonite and POSS-modified silica—in improving the mechanical and flame retardant properties of epoxy-based composites.

## 2. Experimental Section

### 2.1. Materials

Bentonite from Russian deposits, provided by a representative of Bento Group Minerals Company Poland Sp. z o.o., Gdynia, denoted as B.

Epoxy resin Epidian 6—a product of the Organika-Sarzyna chemical plant in Nowa Sarzyna, Poland, denoted as EP.

Triethylenetetramine—a technical product of the Organika-Sarzyna chemical plant Nowa Sarzyna, Poland, denoted as Z-1.

SiO_2_ filler precipitated in polar media, analogously as in the procedure previously described by Jesionowski* et al.* [39]. The silica filler surface (HS) was grafted with POSS modifier (POSS3), in a quantity of 20 parts by weight of SiO_2_.

The bentonite and silica modifiers from the group of cellular silsesquioxanes are presented in Table 1.

**Table 1:** Polyhedral oligomeric silsesquioxanes (POSS) used for modification of bentonite and hydrated silica.

Acronym	Name	Structure
POSS1	octakis(tetramethylammonium) octasilsesquioxane	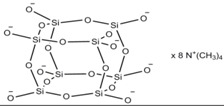
POSS2	octakis {3-(*N*-(hydroxyethyl)dimethylamino) propyl}octasilsesquioxane	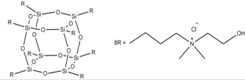
POSS3	Aminoethylaminopropyl isobutyl POSS	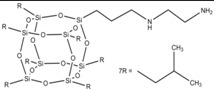

### 2.2. Preparation of Bentonite and SiO_2_ Fillers Modified with Silsesquioxane (POSS)

The study employed the procedure of modification of smectic clays using POSS, which has been previously patented [37,38] and described in the literature [34,35,36].

Silica filler was subjected to functionalization by the method of immobilization in an organic solvent. In a reactor equipped with a high speed stirrer and heating mantle, an appropriate fraction of hydrated silica was introduced into toluene. Nitrogen was introduced to provide an inert atmosphere. When the temperature in the reactor reached 50 °C, the process of immobilization was begun. The modifying mixture was dosed with the use of a peristaltic pump. The mixture was then mixed in the reactor for 2 h (*ca.* 800 rpm). After that time, the organic solvent was separated by distillation. The resulting powder material was dried in a convectional dryer at a temperature of 120 °C for 48 h. The procedure has been described in detail in a previous paper [30].

### 2.3. Determination of Physicochemical Properties of the Fillers

Prior to the determination of particle size, all bentonites were sieved through a 0.06 mm mesh. The test was performed using a Mastersizer Hydro MU2000 apparatus (Malvern Instruments Ltd., Malvern, UK) at 20 °C. The particles of the filler were pre-dispersed ultrasonically in propanol-2-ol for more efficient measurement.

Thermogravimetric analysis of the bentonites was carried out under nitrogen, using a TGA/DSC1 thermobalance (Mettler Toledo, Greifensee, Switzerland). Measurements were made in the temperature range 25–450 °C, with a heating rate of 10 °C/min.

The IR spectra of the bentonites were recorded on a Nicolet 8700 spectrophotometer (Thermo Electron Scientific Instruments LLC, Madison, WI, USA), in the range 4000–4550 cm^−1^, using the KBr pellet technique (1 mg sample/200 mg KBr).

The final silica and SiO_2_/POSS3 hybrid filler were analyzed by a number of methods. The effectiveness and degree of functionalization of SiO_2_ with the POSS compound were estimated using an FTIR IFS 66 v/S spectrophotometer (Bruker Optik GmbH, Ettlingen, Germany). The samples were prepared by mixing with KBr and then pressing into small tablets. FTIR spectra were obtained in the transmission mode between 4000 and 400 cm^−1^.

The structure of the silica surface, before and after modification with POSS, was examined using cross-polarization nuclear magnetic resonance tests. ^29^Si and ^13^C CP MAS NMR measurements were carried out using a DSX spectrometer (Bruker BioSpin GmbH, Rheinstetten, Germany). For the determination of NMR spectra, a sample of about 100 mg was placed in a ZrO_2_ rotator with diameter 4 mm, which enabled spinning of the sample. Centrifugation at the magic angle was performed at a spinning frequency of 8 kHz. ^29^Si CP MAS NMR spectra were recorded at pulse duration 4.5 µs, contact time 1.5 ms, and pulse spacing 6 s. The ^13^C CP MAS NMR spectra were recorded at 100.63 MHz in a standard 4 mm MAS probe using single pulse excitation with high power proton.

The particle size distributions of the silica samples were measured using a Zetasizer Nano ZS (Malvern Instruments Ltd., Malvern, UK), enabling measurements in the range 0.6–6000 nm, by the NIBS method. The microstructures of the samples were analyzed using transmission electron microscopy images (Joel 1200 EX II, JEOL Ltd., Tokyo, Japan).

Thermogravimetric analysis was performed using a Jupiter STA 449 F3 (Netzsch GmbH, Selb, Germany). Samples weighing approximately 10.0 mg were placed in an Al_2_O_3_ crucible, and heated at a rate of 10 °C/min from 30 °C to 1000 °C in a nitrogen atmosphere.

### 2.4. Preparation of Composite of Epoxy Resin Filled with POSS-Modified Silica and Bentonite

The modified bentonite and modified silica prepared in this way, in proportions of 1:1 by weight, were introduced successively in quantities of 1.5–4.5 wt.% to the synthetic resin liquid mixture, and then homogenized in a multistage process. First a modified bentonite was introduced to the epoxy resin, and dispersed until a homogeneous suspension was formed, then modified silica was introduced and dispersed. In both cases, three-stage homogenization was used, based on: (I) pre-mixing by means of a mechanical stirrer at slow rotation at room temperature for 20 min; (II) stirring for 15 min using an ultrasonic homogenizer preheated to a temperature of 50 °C; (III) mixing in a high shear mixer with a turbine stirrer in the vessel, also at 50 °C, with a stirrer speed of 10,000 min^−^^1^, the time of homogenization in the mixer being 30 min; and (IV) final homogenization in a cylindrical vessel with a small gap of 0.75 mm and with the rotary speed of the cylinder equal to 6,000 min^−1^ to provide high shear. The duration of this operation was 15 min.

The composites prepared in this way were stored at about 4 °C to prevent possible sedimentation of the fillers. The specific compositions of the test samples are given in Table 2.

**Table 2:** Composition of test samples based on epoxy resin.

Composite symbol	Content BP1 ^1^, wt.%	Content BP2 ^2^, wt.%	Hybrid content I-HS-P3 ^3^, wt.%
EP	0.0	0.0	0.0
EPBP1-1.5	1.5		-
EPBP1-3.0	3.0		-
EPBP1-4.5	4.5		-
EPIHSP3-1.5	-		1.5
EPIHSP3-3.0	-		3.0
EPIHSP3-4.5	-		4.5
EPBP1IHSP-1.5	0.75		0.75
EPBP1IHSP-3.0	1.5		1.5
EPBP1IHSP-4.5	2.25		2.25
EPBP2-1.5		1.5	-
EPBP2-3.0		3.0	-
EPBP2-4.5		4.5	-
EPBP2IHSP-1.5		0.75	0.75
EPBP2IHSP-3.0		1.5	1.5
EPBP2IHSP-4.5		2.25	2.25

^1^ modified bentonite P1; ^2^ modified bentonite P2; ^3^ modified silica P3.

### 2.5. Obtaining Molded Pieces from the Composites for Structural, Strength and Flammability Tests

Composites based on the resin matrix Epidian 6 were cured using Z-1 (13 wt.%), according to the resin manufacturer’s instructions. The composites were then vented in a Vakuum UHG 400 laboratory vacuum chamber (Schuechl, Bawaria, Germany) and cast at 40 °C in silicone molds prepared in accordance with ISO 527-1:1998. The molded pieces were cured at room temperature for 24 h and then post-cured at 100 °C for 6 h. After two days, the molded pieces were tested in accordance with the relevant standards.

### 2.6. Study of Mechanical Properties of Composites

Tensile strength and Young’s modulus were determined according to ISO 527-1:1998 using an INSTRON 5967 testing machine equipped with an advanced videoextensometer. “Dog-bone” specimens (type 2 according to the standard) were used. Young’s modulus was obtained as the tangent to the linear segment of the stress-elongation curve. A rate of elongation of 2 mm/min was used for the linear stress-elongation curve segment, and after that 20 mm/min was used. The measurement temperature was 23 °C.

Charpy impact strength was determined according to DIN EN ISO 179-1 with a PSW4J camera (Gerhard Zorn, Berlin, Germany), using a hammer impact energy of 1 J with a digital result readout. Unnotched bars: 100 mm long, 10 mm width and 4 mm height were used.

Rockwell hardness was measured using a Zwick 3106 durometer (Zwick/Roell, Ulm, Germany), in accordance with EN 10109-1. The applied indenter load was equal to 358 N. Similar specimens as for Charpy impact testing were used.

The measurement temperature used for mechanical tests was 23 °C. All final results given for the mechanical properties of the studied composites are mean values from 10 specimen tests. Standard deviations were also calculated.

### 2.7. Study of the Morphology and Structure of Composites

The brittle fracture morphology of the composites was analyzed using scanning electron microscopy (SEM 234a, JEOL Ltd., Tokyo, Japan). Fractured profiles were obtained after cooling in dry ice and impact-break. The microstructure of the composites was also observed using a Tecnai G2 SpiritTwin type 12 transmission electron microscope (TEM) (FEI Company, Hilsboro, NE, USA) at an accelerating voltage of 120 kV. Ultra-thin cuttings were performed at room temperature using a Tesla ultramicrotome (Tescan Orsay Holding, a.s, Brno, Czech Republic) with glass knives. They were collected on the surface of 10% aqueous acetone and placed on standard microscope copper grids.

Imaging of the surface of the samples was performed using an atomic force microscope (AFM) (Bruker Nano Surfaces Division, Santa Barbara, CA, USA) by the QNM technique. On the basis of local changes in the Young’s modulus of the sample surface, rigid areas that correspond to the presence of fillers, and areas with less rigidity associated with the polymer matrix, were observed. This enabled estimation of the degree of homogenization of the filler on the surface of the polymer matrix, and of the size of its particles. The tests were performed using a Nanoscope V microscope (Bruker Nano Surfaces Division, Santa Barbara, CA, USA) with an RTESPA scanning needle, with a resonance frequency of 270 kHz. The scanning speed was 1 kHz, and the resolution 256 lines.

IR maps of the surface of the composites were made using a Nicolet FTIR MX IN10 microscope (Thermo Electron Scientific Instruments LLC, Madison, WI, USA), to determine the intensity of distribution of the characteristic Si–O–Si functional groups. This served to assess the uniformity of dispersion of grains of the filler in the composite.

The separation of plates in bentonites and their EP composites was assessed by wide-angle X-ray scattering (WAXS), using Braggs’ law [40]. The measurements were performed using a Bruker Nanostar diffractometer (Bruker AXS, Inc. Madison, WI, USA) with Cu lamp, for the bandwidth Kα. The samples were in the form of disks 25 mm in diameter and 2 mm in thickness, cast from the tested composites. The bentonite samples were tested in powder form.

### 2.8. Study of Flammability of Composites

Determination of the oxygen index (LOI) was performed at 25 °C according to EN ISO 4589-3, using apparatus made by Fire Testing Technology Ltd. (East Grinstead, UK).

UL 94 flammability testing was performed in a chamber designed for such tests (Fire Testing Technology Ltd., East Grinstead, UK). The measurements were made in accordance with PN-EN 60695-11-10.

Morphological and elemental analysis of burnt composite samples was performed using a Hitachi S-3400N scanning electron microscope (SEM) (Tokyo, Japan) equipped with an adapter for microanalysis (EDS) of chemical composition. Measurements were performed using a detector of secondary electrons (SE) (Thermo Scientific, Karlsruhe, Germany) and backscattered electrons (BSE) (Thermo Scientific, Karlsruhe, Germany), with an accelerating voltage of 15 kV and spot size <10 nm.

### 2.9. Determination of Gelation Time of the Hybrid Composites

Gelation time after the addition of Z-1 hardener was measured at 25 °C according to PN-EN ISO 2535, using a WB-2 gel penetrator device (Rzeszow University of Technology, Rzeszow, Poland) of our own design and construction. The apparatus enables the tracking of temperature changes during the curing of a reaction mixture.

## 3. Results and Discussion

### 3.1. Analysis of the Process of Modification of Bentonites

**Figure 1:**
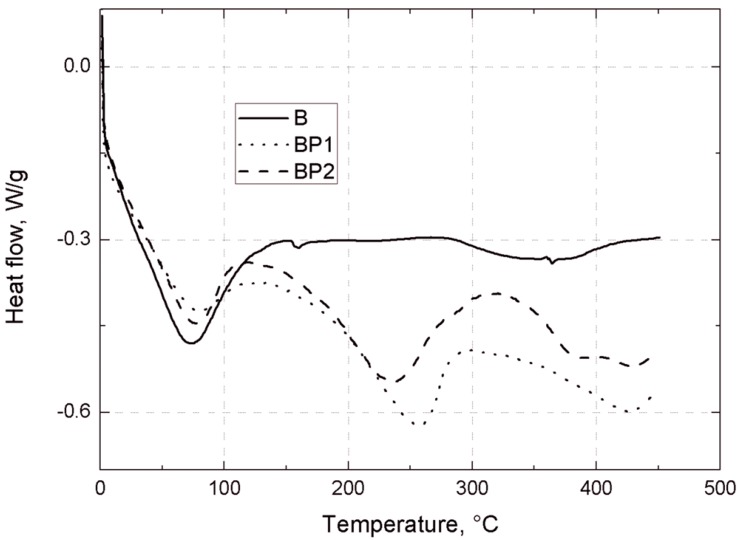
DSC curves of the examined bentonites: unmodified bentonite (B), POSS1-modified bentonite (BP1) and POSS2-modified bentonite (BP2).

**Figure 2:**
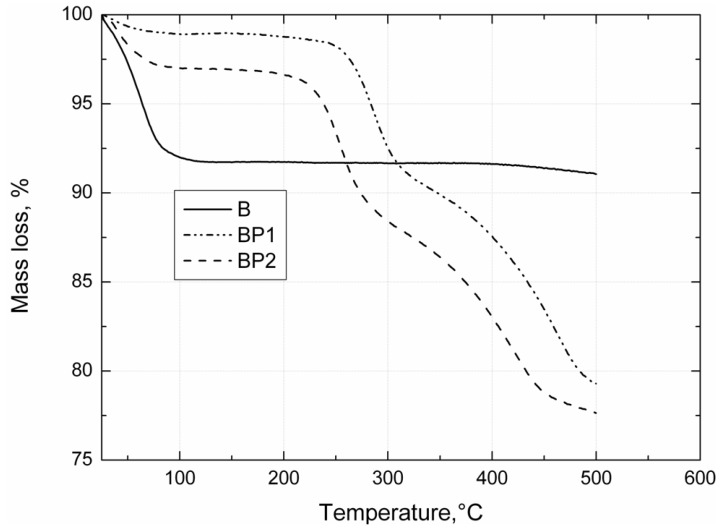
TGA curves of the bentonites B, BP1 and BP2.

DSC curves for unmodified bentonite (B), POSS1-modified bentonite (BP1) and POSS2-modified bentonite (BP2) are shown in Figure 1. On the DSC curves for BP1 in the temperature ranges 240–280 °C and 400–430 °C, and for BP2 in the ranges 225–260 °C and 380–440 °C, two distinct endothermic peaks are visible. These effects are related to the collapse of the alkyl substituents of the POSS modifier built into the structure of the clay [41], which is accompanied by a marked weight loss, visible on the TGA curve in Figure 2. On the DSC and TGA curves of unmodified bentonite (B), no such changes are observed. On the DSC curve of the filler B there is an endothermic peak in the range 70–130 °C (shown in Figure 1), and the corresponding weight loss of the sample, visible on the TGA curve in Figure 2, is related to loss of moisture [42]. Similar effects of slightly lower intensity also exist for BP1 and BP2, but only in the range 80–100 °C.

**Figure 3:**
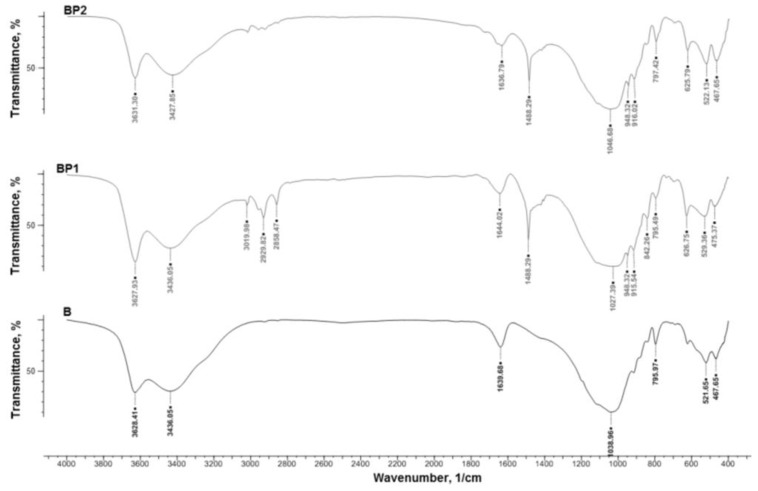
IR spectra of unmodified bentonite (B) and the modified (functionalized) bentonites BP1 and BP2.

To gain further information on the effectiveness of the modification of bentonite, IR spectroscopy was performed to study unmodified B, BP1, and BP2, as shown in Figure 3. The FTIR spectra of BP1 and BP2 bentonites showed a peak at wavenumber 1488.3 cm^−1^, derived from the –NH_3_^+^ group [43], which is not present in the spectrum of unmodified bentonite. In the IR spectrum of BP1 bentonite, there are, additionally, three peaks at wavenumbers 2929.8 cm^−1^ and 2858.5 cm^−1^, which are linked to the asymmetric and symmetric stretching vibrations of C–H bonds present in methyl groups in the silsesquioxane modifier (Table 1).

**Figure 4:**
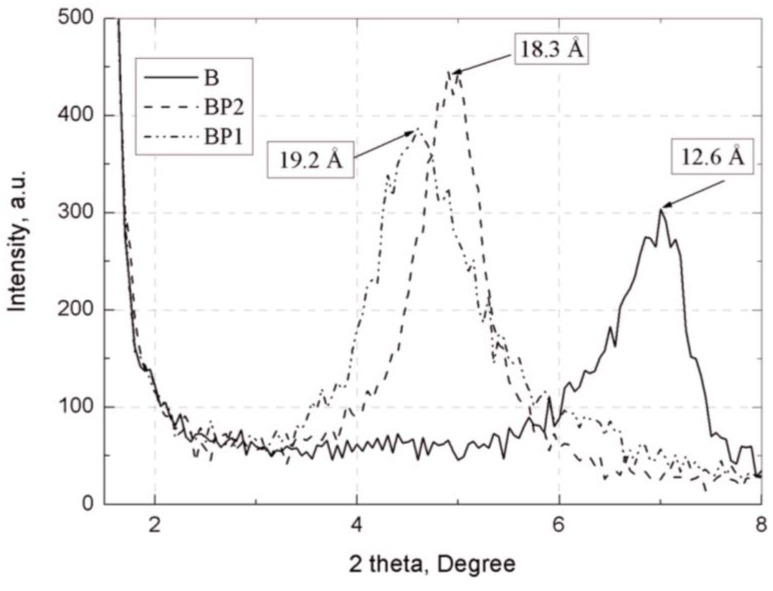
WAXS curves: unmodified bentonite (B) and samples BP1 and BP2 (d_001_) expressed in Å.

Confirmation of the effectiveness of the modification process (separation of plates of bentonite B) was provided by the X-ray studies (see Figure 4). On the basis of WAXS curves, it was found that the distance between the plates (d_001_) of bentonite B modified with POSS1 and POSS2 (see Table 1) increased markedly, from 12.6 Å for B to about 19.2 Å for BP1 and 18.3 Å for BP2. A greater d_001_ value facilitates the migration of polymer chains between the layers of the filler, which favors the dispersion of the aluminosilicate plates to nanometric size.

### 3.2. Analysis of Grain Size Distribution of Modified Bentonites

For the modified bentonites obtained as described above, the grain size distribution (granulation) was investigated. The results are shown in Table 3.

**Table 3:** Summary of results for particle size distribution of bentonite, unmodified and modified with POSS1 and POSS2.

Bentonite symbol	Grain size distribution, %				
	0.5–1 μm	1–5 μm	5–10 μm	10–50 μm	50–100 μm
B	4.1	13.4	20.4	30.6	31.5
BP1	3.5	10.6	18.3	33.2	34.4
BP2	2.6	9.3	17.4	31.8	38.9

Based on the test results, some differences in grain size were observed between the unmodified bentonite (B) and bentonite modified with POSS1 (BP1) or POSS2 (BP2). For BP1 and BP2, there is an increase in the size of the 10–50 µm and 50–100 µm fractions. It is, therefore, noted that the bentonites used to fill polymers have micron-sized grains, and only after their dispersion in resin by appropriate multiple-stage homogenization are they reduced to nanosize.

### 3.3. Assessment of the Modification Process of the Hybrid System: FTIR, ^29^Si and ^13^C CP MAS NMR Analysis

**Figure 5:**
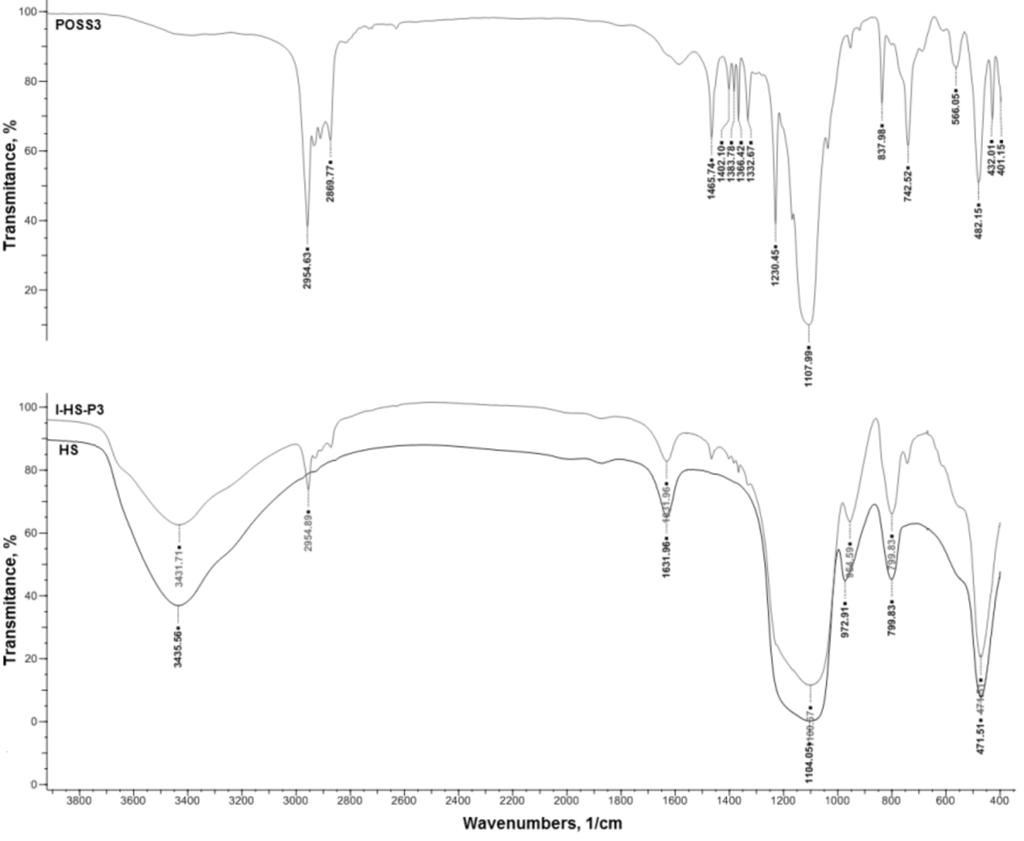
FTIR spectra of pure aminoethylaminopropylisobutyl POSS (POSS3) (**a**), unmodified hydrated silica (HS), and a hybrid prepared on its basis, grafted with 20 wt.% of N POSS (I-HS-P3) (**b**).

The interactions between the components of the SiO_2_/POSS3 hybrid system were assessed by FTIR measurements. In Figure 5a,b, FTIR spectra of pure aminoethylaminopropylisobutyl POSS (POSS3), unmodified hydrated silica (HS), and a hybrid filler prepared on its basis grafted with 20 parts by weight of POSS3 (I-HS-P3), are shown.

**Figure 6:**
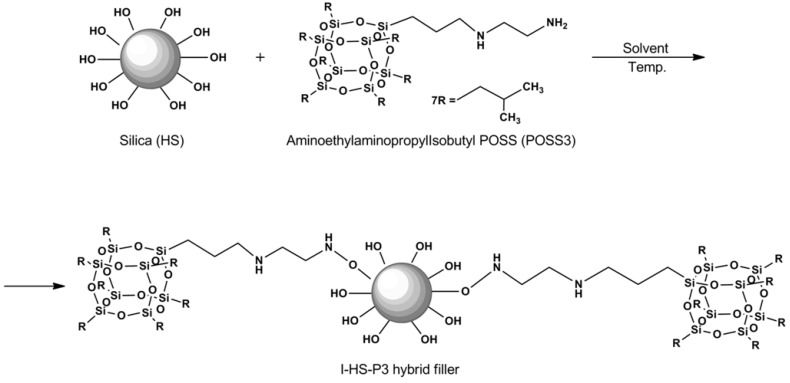
Predicted mechanism of silica surface functionalization.

In the FTIR spectrum of pure aminoethylaminopropylisobutyl POSS (POSS3), a wide bandwidth, attributed to stretching vibrations of −NH groups, was observed in the wavenumber range 3500−3200 cm^−1^. Additionally, an intense band attributed to stretching vibrations of −CH in the range 3000−2900 cm^−1^, and a peak at 1640 cm^−1^, were attributed to vibrations in molecules of physically absorbed water [44,45]. In the spectrum of the unmodified POSS compound (POSS3, Figure 5) there are bands attributed to flexural vibrations of the −NH groups derived from a primary amine, at 1695 cm^−1^; to bending vibrations of −NH derived from a secondary amine, at 1450 cm^−1^; and to −CH bending vibrations, at 1300 cm^−1^. Additionally shown in Figure 6a is an absorption band characteristic of stretching and bending vibrations of Si−O–Si at 1100 cm^−1^, while at 800 cm^−1^ a band characteristic of stretching vibrations of Si–OH was observed.

In the spectrum of the hydrated silica (HS, Figure 5), a broad band is observed in the range 3600–3200 cm^−1^, attributed to ν (Si–OH), as well as two characteristic absorption bands at 1100 cm^−1^ and 495 cm^−1^, attributed to the stretching vibrations of ν (Si–O–Si), and a peak at 1640 cm^−1^ attributed to the vibration of physically absorbed water molecules [44,45]. As a result of functionalization of the surface of the silica support, besides the characteristic bands typical for silica, there is also a strong absorption band at 1450 cm^−1^ attributed to flexural vibrations of −NH groups derived from a secondary amine, and an absorption band of ν (−CH) at 1300 cm^−1^. Moreover, a ν (−NH) band at 3250 cm^−1^, which is effectively masked by the ν (Si−OH) band [30,46], was observed. The proposed mechanism of interaction between the hydroxyl groups of the silica and the reactive group of the aminoalkyl is shown in Figure 6.

**Figure 7:**
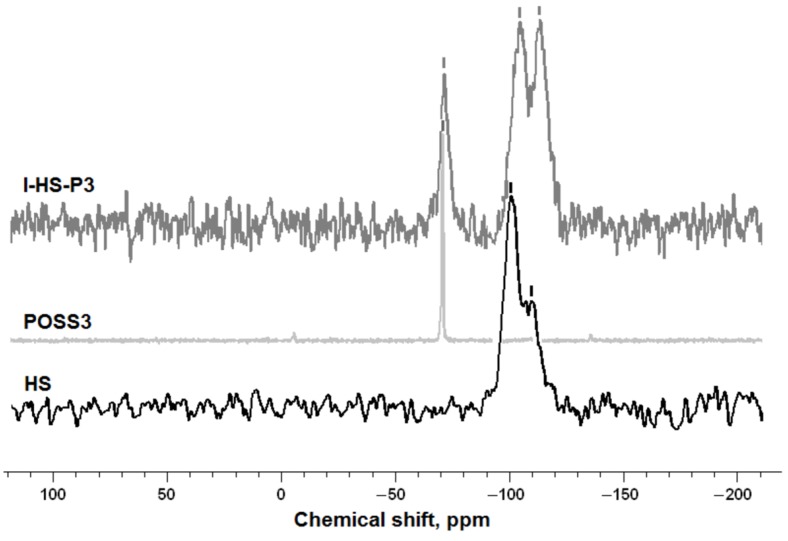
^29^Si CP MAS NMR spectra of unmodified hydrated silica (HS), pure aminoethylaminopropylisobutyl POSS (POSS3), and SiO_2_ grafted with POSS3 (I-HS-P3).

Figure 7 shows the ^29^Si CP MAS NMR spectra of the unmodified silica support, pure aminoethylaminopropylisobutyl POSS (POSS3), and SiO_2_ functionalized with POSS3 (I-HS-P3). This NMR method also served to determine the nature of the interactions taking place between the support and modifier.

The ^29^Si CP MAS NMR spectrum of the silica support shows two signals, at −100 and −110 ppm. The main signal at −100 ppm is attributed to a structure of three siloxane groups and one silanol group (isolated silanols—Q_3_). The second signal at −110 ppm is attributed to four siloxane bridges (Q_4_). After treatment of the silica surface with aminoethylaminopropylisobutyl POSS, the Q signals are changed. This could be caused by the loss of hydroxyl groups and the formation of ≡Si–O–Si≡ linkages, which is demonstrated by the change in the intensity of the Q signals in the ^29^Si CP MAS NMR spectrum. In the spectrum of hydrated silica after the functionalization process, a T^2^ structure was observed at −67 ppm, corresponding to R_2_Si(O_0.5_)_2_ [47,48]. This provides evidence of the chemical interactions of the modifier and of the effectiveness of the modification. 

Further proof of the chemisorptions of the modifier is provided by the ^13^CP MASNMR analysis. This analysis was performed for pure aminoethylaminopropylisobutyl POSS (POSS3), and for the hybrid obtained when SiO_2_ was functionalized with POSS3 (I-HS-P3).

The formation of the products were verified by ^13^C NMR analysis (C_6_D_6_, 100.63 MHz, ppm): **POSS3**: 21 (Si–CH_2_–); 24.0 (–CH_2_–); 41 (–CH_2_–NH_2_); 50 (C–NH–), **I-HS-P3**: 21 (Si–CH_2_–); 50 (C–NH–); this is analogous to [29,49].

### 3.4. Dispersive and Morphological Characteristics

For the non-modified silica support and hybrids produced on its basis, modified with 20 parts by weight of POSS3, morphological dispersion analysis was performed to assess the possible degree of agglomeration.

### 3.5. Thermal Analysis

**Figure 8:**
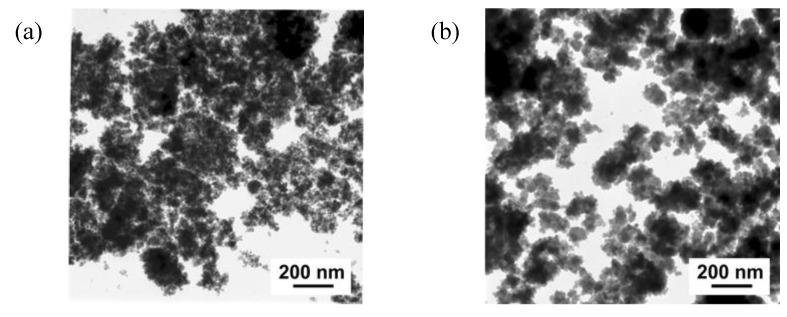
TEM microphotograph of unmodified SiO_2_ (**a**) and silica functionalized with 20 parts by weight of POSS3 (**b**).

As Table 4 shows, there is some variation in particle size between the unmodified silica (HS) and POSS3-modified silica (I-HS-P3). The 50–150 nm and 4000–6500 nm fractions are larger in HS. Additionally, the TEM image (Figure 8a) shows that the precipitated silica has a tendency to form primary and secondary agglomerates. The polydispersity index of the resulting support is 0.884. Table 4 presents the particle size distribution of the resulting hybrid system with silica functionalized with 20 parts by weight of aminoethylaminopropylisobutyl POSS (POSS3). For sample I-HS-P3, there was an increase in the 150–250 nm fraction, corresponding to primary particles, and in the 4000–5000 nm fraction, originating from secondary particles; this fraction accounts or the largest share of the volume (41.4%).

**Table 4:** Summary of results for grain size distribution of unmodified silica (HS) and silica modified with POSS 3 (I-HS-P3).

Acronym	Grain size distribution of the filler, %				
	50–150 nm	150–250 nm	3000–4000 nm	4000–5000 nm	5000–6500 nm
HS	28.5	16.1	7.3	26.4	21.7
I-HS-P3	-	32.4	10.9	41.4	15.3

It can be seen in Table 4 that the introduction of 20 parts by weight of POSS3 modifier to the surface of SiO_2_ resulted in a slight change in the dispersion properties of the finished product when compared with the original support. In addition, the TEM microphotograph in Figure 8b indicates the presence of significant quantities of primary particles. Larger clusters of particles and varying degrees of homogeneity have not been eliminated entirely, as is confirmed by the polydispersity index value of 0.615.

**Figure 9:**
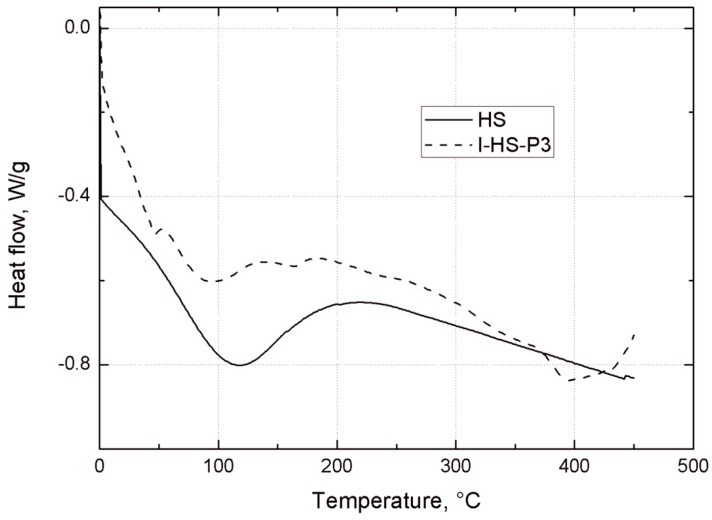
DSC curves for the studied bentonites: B, BP1, and BP2.

**Figure 10:**
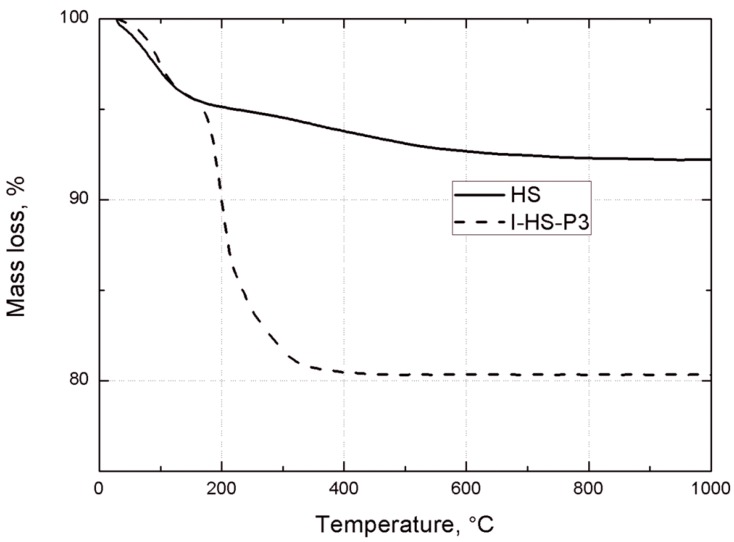
TGA curves of unmodified silica HS, and POSS3-modified silica I-HS-P3.

Figure 9 shows the DSC curves of unmodified silica (HS), and silica modified with POSS3 (I-HS-P3). On the DSC curve for I-HS-P3 in the temperature ranges 240–280 °C and 400–430 °C, and for BP2 in the range 390–440 °C, there are clear endothermic peaks. This effect is related to the dissolution of the POSS modifier embedded in the structure of the clay [30,50], which is accompanied by marked weight loss, observed on the TGA curve in Figure 10. On the DSC and TGA curves for unmodified silica (HS), no such changes were observed. However, on the DSC curve of the HS filler, an endothermic peak is present in the range 60–150 °C (Figure 9), and there is a corresponding sample weight loss shown on the TGA curve, related to the loss of moisture [51]. A similar effect, with slightly lower intensity, is also found for I-HS-P3, but in the range 50–130 °C.

### 3.6. Effect of Bentonite Nanofillers on the Gelation Time of Epoxy Resin

**Figure 11:**
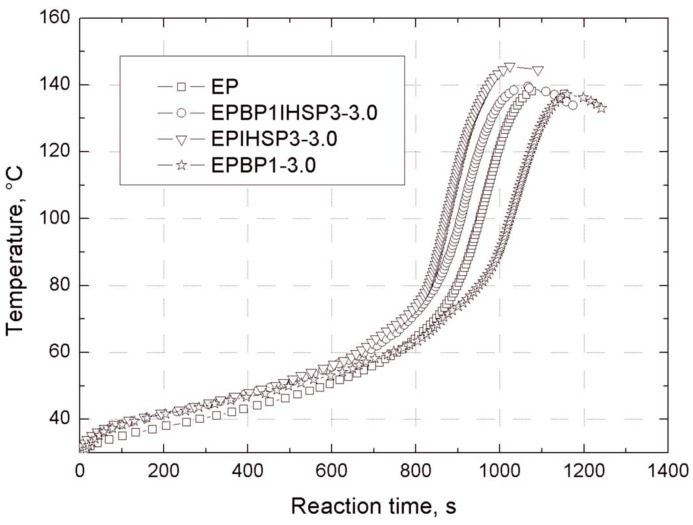
Gelation curves of epoxy resin (EP) and the composites EPPOSS1IHSP3-3.0, EPIHSP3-3.0 and EPBP1-3.0.

Based on the results presented in Table 5 and Figure 11, it was found that the addition of silica to the EP hybrid system reduces gelation time by about 8%, and increases the maximum curing temperature by up to 10 °C. The presence of the modified bentonite BP1 or BP2 in the epoxy resin prolongs the gelation time by 7%–16%, with an increase in the concentration of the bentonite in the composite. The maximum crosslinking temperature is practically unchanged (see Table 5 and Figure 11). However, the addition of the modified silica to a composite containing modified bentonite reduces its gelation time by approximately 5%.

**Table 5:** Results for gelation parameters (±standard deviation).

Composite symbol	Gelation time, s	Maximum crosslinking temperature, °C	Glass temperature, °C
EP	244 ± 5	135.3 ± 0.2	108.0
EPIHSP3-1.5	243 ± 2	136.2 ± 0.2	
EPIHSP3-3.0	223 ± 2	144.9 ± 0.4	110.0
EPIHSP3-4.5	220 ± 3	145.3 ± 0.3	
EPBP1-1.5	261 ± 2	134.3 ± 0.3	
EPBP1-3.0	268 ± 3	132.9 ± 0.6	109.5
EPBP1-4.5	284 ± 4	126.3 ± 0.4	
EPBP1IHSP3-1.5	255 ± 4	136.6 ± 0.2	
EPBP1IHSP3-3.0	255 ± 4	136.6 ± 0.2	119.5
EPBP1IHSP3-4.5	273 ± 3	131.2 ± 0.5	
EPBP2-1.5	263 ± 3	134.5 ± 0.2	
EPBP2-3.0	268 ± 3	133.3 ± 0.4	109.0
EPBP2-4.5	285 ± 4	126.7 ± 0.5	
EPBP2IHSP3-1.5	256 ± 4	137.1 ± 0.3	
EPBP2IHSP3-3.0	255 ± 3	136.9 ± 0.3	111.5
EPBP2IHSP3-4.5	274 ± 3	131.9 ± 0.4	

The introduction of the nanofillers BP1, BP2 and IHSP3 into the epoxy matrix caused a slight increase in the glass temperature of the epoxy composites (by ~1–2 °C), as shown in Table 5. The largest increase in the glass temperature of the composite (up to 119.5 °C) came from the hybrid BP1IHSP3 nanofiller (Table 5). This nanofiller is composed of modified bentonite and POSS-modified silica.

### 3.7. Mechanical Properties of EP Composites Filled with Modified Bentonites

#### 3.7.1. Tensile Strength

Test results for a dumbbell cast of EP composites, comprising a combination of the hybrid fillers BP1, BP2 or I-HS-P3, used in quantities of 1.5–4.5 wt.%, are shown in Table 6.

**Table 6:** Mechanical and flammability properties of tested epoxy composites (±standard deviation).

Composite symbol	Tensile strength, MPa	Young’s modulus, GPa	Charpy impact strength, kJ/m^2^	Rockwell hardness, MPa	LOI, %	Flammability class according to UL 94
EP	46.9 ± 1.1	2.99 ± 0.23	4.21 ± 0.49	144.9 ± 1.2	19.0 ± 0.3	flammable
EPIHSP3-1.5	52.8 ± 1.2	3.14 ± 0.18	4.65 ± 0.63	156.8 ± 1.9	21.4 ± 0.6	HB
EPIHSP3-3.0	63.8 ± 0.6	3.31 ± 0.09	6.18 ± 0.24	167.5 ± 1.1	23.3 ± 0.4	HB
EPIHSP3-4.5	64.2 ± 0.7	3.42 ± 0.16	5.91 ± 0.61	171.3 ± 1.3	23.8 ± 0.3	HB
EPBP1-1.5	50.9 ± 0.9	3.09 ± 0.12	5.89 ± 0.36	142.1 ± 1.1	24.6 ± 0.2	V2
EPBP1-3.0	62.4 ± 0.8	3.24 ± 0.14	7.83 ± 0.37	139.5 ± 2.1	28.1 ± 0.2	V1
EPBP1-4.5	63.1 ± 1.1	3.31 ± 0.11	7.42 ± 0.84	138.2 ± 2.4	26.4 ± 0.4	V2
EPBP1IHSP3-1.5	54.7 ± 1.3	3.17 ± 0.11	6.11 ± 0.33	143.9 ± 1.6	24.8 ± 0.3	V2
EPBP1IHSP3-3.0	66.9 ± 0.9	3.43 ± 0.12	8.12 ± 0.24	153.8 ± 1.9	29.2 ± 0.3	V1
EPBP1IHSP3-4.5	67.6 ± 1.2	3.45 ± 0.13	7.67 ± 0.41	156.4 ± 1.1	26.8 ± 0.5	V2
EPBP2-1.5	48.6 ± 0.9	3.03 ± 0.09	5.60 ± 0.29	142.2 ± 1.2	23.5 ± 0.1	V2
EPBP2-3.0	59.9 ± 0.9	3.18 ± 0.11	7.32 ± 0.32	140.1 ± 1.4	26.9 ± 0.2	V2
EPBP2-4.5	60.6 ± 1.0	3.25 ± 0.09	6.65 ± 0.56	138.9 ± 1.7	26.2 ± 0.2	V2
EPBP2IHSP3-1.5	52.2 ± 1.2	3.11 ± 0.08	5.91 ± 0.34	144.3 ± 1.3	23.8 ± 0.2	V2
EPBP2IHSP3-3.0	64.4 ± 0.8	3.37 ± 0.10	7.34 ± 0.24	154.1 ± 1.2	27.6 ± 0.1	V1
EPBP2IHSP3-4.5	65.1 ± 1.1	3.39 ± 0.11	7.46 ± 0.53	156.6 ± 1.2	26.4 ± 0.2	V2

The addition of POSS1-modified bentonite to EP resulted in an increase in the tensile strength and Young’s modulus of the composites. With an increase in the content of BP1 in the composite, the ultimate tensile strength increased by approximately 35%, and the Young’s modulus by approximately 11% (Table 6). It was found that a 4.5% concentration of the filler in the composite EP + BP1 made no improvement to the ultimate tensile strength and Young’s modulus, as compared with samples where the filler content was 3%. This may show that a certain limit of concentration of the modified bentonite in the EP matrix has been reached. Much poorer results were obtained for the breaking stress and Young’s modulus for EP matrix composites with the addition of BP2. Increasing concentrations of BP2 did not bring about further marked improvement in the strength characteristics (Table 6). This was connected with the tendency for the formation of an intercalated, not exfoliated, structure of the filler in the matrix of the resin (confirmed by the WAXS results shown in Figure 15). However, in the case of EP composites containing 1.5–4.5 wt.% of POSS-modified silica (I-HS-P3), the breaking stress values obtained are slightly less when compared with the composites containing modified bentonite shown in Table 6. In this case, a significantly greater improvement can be seen in the Young’s modulus. The best results were obtained with a 4.5% content of the filler in the composite.

Significant improvement in the ultimate tensile strength and Young’s modulus can however be observed for composites containing both hybrid fillers: bentonite modified with POSS1 or POSS2 and modified silica, with a 1:1 weight ratio. It was found that with increasing contents of both hybrid fillers in the composite, the breaking stress increased by approximately 44% and the Young’s modulus by approximately 15%, as shown in Table 6. It was found that the most favorable content of the two fillers in the resin is 3% (1.5% BP1 and 1.5% I-HS-P3). Comparison of the two types of modified bentonite shows that a greater improvement in ultimate tensile strength and Young’s modulus was obtained for composites containing BP1 and I-HS-P3 than for those containing BP2 and I-HS-P3.

#### 3.7.2. Rockwell Hardness

The results summarized in Table 6 indicate that the hardness of the composites was affected by the concentration of the modified silica and modified bentonite and by the nature of the POSS used to modify it. The results show that, when BP1 and BP2 are used for filling EP, measured Rockwell hardness slightly decreased as compared with the unfilled resins. The greatest decrease in this parameter was observed for the composites with an EP matrix containing BP1, for which it decreased by 2%–5% with increasing filler content in the resin. For composites of EP containing BP2, Rockwell hardness decreased by 2%–4% (Table 6). These results indicate that the EP resin becomes more flexible under the influence of the modified bentonite used in the study. However, for composites containing modified silica (I-HS-P3), a marked improvement in Rockwell hardness, by 8%–18% with increasing filler content in the resin, was observed. As one would expect, the addition of silica to the hybrid composite containing BP1 or BP2 did not cause the Rockwell hardness to deteriorate compared with the uninflated resin. It was also found that, with a 3% or 4.5% content of filler in the composite, the hardness showed a slight increase, by 6%–8%.

#### 3.7.3. Charpy Impact Strength

The data presented in Table 6 show that the filling of EP with bentonite B modified with POSS1 significantly increased the impact strength of the composites. Much worse impact test results were obtained for EP composites with the addition of BP2. In the case of composites containing the modified silica additive (I-HS-P3), an improvement was observed in the impact strength of uninflated resin, but not as large as in the case of composites containing the modified bentonites (Table 6). The best results were obtained for composites containing both modified silica and modified bentonite. It should be noted that a large role is played by their concentration in the composite, and among the samples tested, the largest increase in impact strength compared with the unmodified sample was observed for the composite EP + 1.5% + 1.5% BP1 I-HS-P3 (EPBP1IHSP3-3.0). It should be noted, however, that the impact strength of the composites EP + 2.25% BP1 I-HS-P3 (EPBP1IHSP3-4.5) was lower by 10 kJ/m^2^. This is consistent with the conclusion that a content of 3% of both fillers in the composite seems to be the economically optimal concentration in terms of improving the mechanical properties.

### 3.8. Morphology of the Hybrid Composites

**Figure 12:**
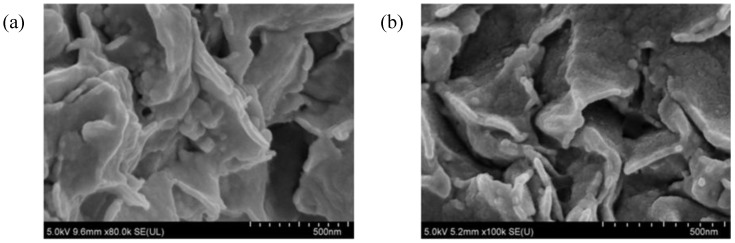
SEM microphotographs of brittle fractures: EP with the addition of 1.5% BP1 and 1.5% I-HS-P3 (EPBP1IHSP3-3.0) (**a**); and EP with the addition of 2.25% BPOSS and 2.25% I-HS-P3 (EPBP1IHSP3-4.5) (**b**).

**Figure 13:**
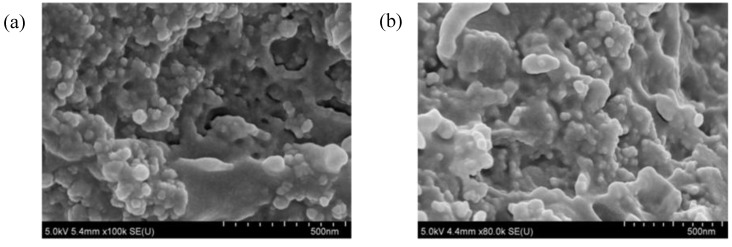
SEM microphotographs SEM of brittle fractures: EP with the addition of 3.0% I-HS-P3 (EPIHSP3-3.0) (**a**); and EP with the addition of 4.5% I-HS-P3(EPIHSP3) (**b**).

On the basis of the SEM microphotographs of brittle fractures of the tested molded pieces, shown in Figure 12, it is observed that the nanofillers used to fill the resin (BP1 bentonite and modified silica) disperse evenly. In the fracture of the EP composite with the addition of 1.5% BP1 and 1.5% I-HS-P3, there are clear aluminosilicate plates ranging in size from 250 to 400 nm, with silica grains among them. Such a fine-plate composite structure is typical for nanocomposites. Unfortunately, an increase of the content of the fillers in the composite from 3 to 4.5 wt.% (EP with the addition of 2.25% BPOSS and 2.25% I-HS-P3) reduces the distance between the plates, and in addition, on the surface plates of the modified bentonite, rough protuberances are formed, which are likely to be agglomerates of silica, significantly impairing the useful properties of composites containing BP1 (Figure 12b). Microscopic examination (SEM) of the EP composites containing BP2 gave similar results. For the composites EPIHSP3-3.0 and EPIHSP3-4.5, the formation of agglomerates of the filler in the resin can be observed in Figure 13a and 13b.

**Figure 14:**
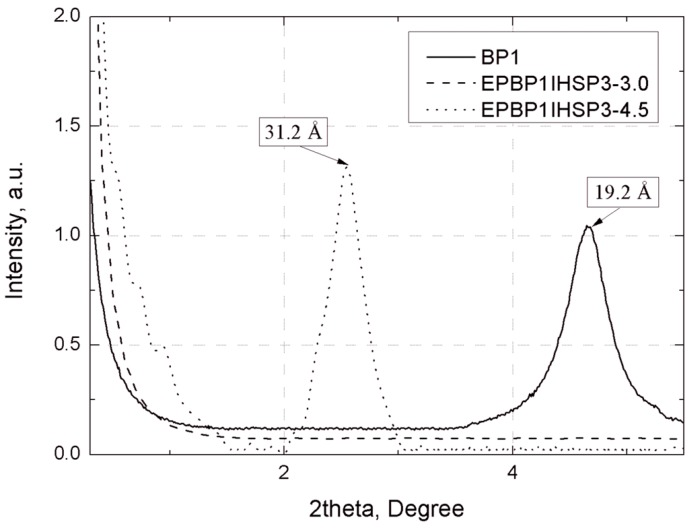
WAXS curves of BP1 and the composites EPBP1K-3.0 and EPBP1K-4.5.

**Figure 15:**
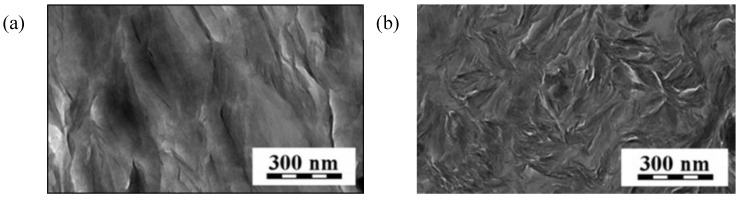
TEM microphotographs of ultra-microtome cuttings of nanocomposites: EPBP1IHSP3-3.0 (**a**), EPBP1IHSP3-4.5 (**b**).

The WAXS diffraction patterns (Figure 14) of the composites EPBP1IHSP3-3.0 show that the current diffraction maximum characteristic for the output aluminosilicate (BP1) vanishes. This indicates the possible formation of an exfoliated structure in these composites,* i.e.*, a structure with full extension and dispersion of the bentonite plates in the polymer matrix [52]. For a 4.5% concentration of filler in the composite (EPBP1IHSP3-4.5) the characteristic peak for the occurrence of plate galleries in clay was observed, although the distance between the plates is greater than 15 Å with respect to the corresponding distance in BP1 (Figure 4). This may indicate the formation of an intercalated structure, in which the polymer penetrates into the plate galleries of modified aluminosilicate. The WAXS curves of EP composites with the addition of BP2 and modified silica show the formation of intercalated structures, and the characteristic peak for BP2 occurring at 18.3 Å (Figure 15) shifted to 31.2 Å for EPBP2IHSP3-3.0 and to 27.4 Å for EPBP2IHSP3-4.5. The smaller value of the bentonite plate separation in the composite EPBP2IHSP3-4.5 is probably caused by agglomeration of the fillers, which indicates that its most favorable content in the composite is ~3%. On the basis of the WAXS diffraction maxima and Scherrer’s formula [53], bentonite plate size was calculated before and after addition of the filler to the composite (Table 7).

**Table 7:** Plate sizes and distances between plates for the studied bentonites and composites.

Composite or filler symbol	Distance between bentonite plates (d_001_), Å	Size of filler plates, Å
B	12.6	235
BP1	19.2	225
BP2	18.3	220
EPBP1IHSP3-1.5	∞	*
EPBP1IHSP3-3.0	∞	*
EPBP1IHSP3-4.5	34.2	210
EPBP2IHSP3-1.5	∞	*
EPBP2IHSP3-3.0	31.2	200
EPBP2IHSP3-4.5	27.4	195

* The plate size was not calculated because no maximum was recorded on the WAXS curve, this being characteristic for nanocomposites with exfoliated structure.

The calculated sizes of the bentonite plates, summarized in Table 7, indicate that after the modification process with P1 and P2, there is a slight decrease in plate size from 235 Å for B to 225 Å for BP1 and 220 Å for BP2. There is also a small reduction (by about 10 Å) in the size of plates of aluminosilicate in the EPBP1IHSP3-4.5, EPBP2IHSP3-3.0, and EPBP2IHSP3-4.5 nanocomposites with intercalated structure. These results indicate that during the modification of bentonite by silsesquioxanes, the mineral plates practically do not change in size, which results in composites with better mechanical properties. In the case of composites EPBP1IHSP3-1.5, EPBP1IHSP3-3.0, and EPBP2IHSP3-1.5, estimation of the size of the plates was not possible due to the absence of a WAXS maximum in the curve, which is characteristic for nanocomposites with an exfoliated structure [54,55].

The TEM microphotographs of ultra-thin cuttings of molded pieces confirm the observation from the WAXS analysis that the addition of 1.5% BP1 and 1.5% I-HS-P3 to EP (EPBP1IHSP3-3.0) leads to a nanocomposite with exfoliated structure (Figure 15a). Each of the plates visible in the photomicrograph is isolated from the other, and there is no obvious presence of agglomerates. In the case of composites with a 4.5% filler content (EPBP1IHSP3-4.5), complete exfoliation of the fillers was not observed, yet areas with intercalated structure can be seen (Figure 15b). TEM microphotographs of composites with both 3% and 4.5% filler content (EPBP2IHSP3-3.0, and EPBP2IHSP3-4.5) show no exfoliation of the fillers; in both cases, a nanocomposite with intercalated structure was obtained.

**Figure 16:**
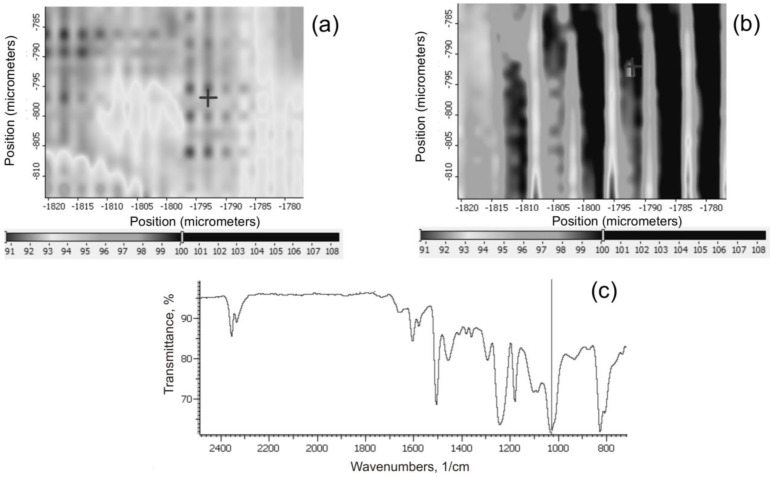
IR maps showing the position of the 1040 cm^−1^ band characteristic of Si–O–Si groups, in the composites: (**a**) EPBP1IHSP3-3.0; (**b**) EPIHSP3-3.0; (**c**) composite spectrum with marked Si-O-Si band. +, sample surface place on which spectrum (**c**) was recorded.

The maps of selected surface areas of composite samples recorded by the IR microscope make it possible to evaluate the degree of dispersion of modified aluminosilicates and silica by measuring the intensity of the 1045 cm^−^^1^ band characteristic of Si–O–Si bonds. In the case of the composites EPIHSP3-3.0, well dispersed agglomerates of grains of modified silica are seen in the polymer matrix (Figure 16b)—clear, bright bands with a gray border are observed. For the composites obtained with the addition of both fillers (EP BP1 IHSP3-3.0, Figure 16a), there is an increase in the intensity of bands that coincide with the bands of modified bentonite [19]—the clear bright bands visible in the image.

**Figure 17:**
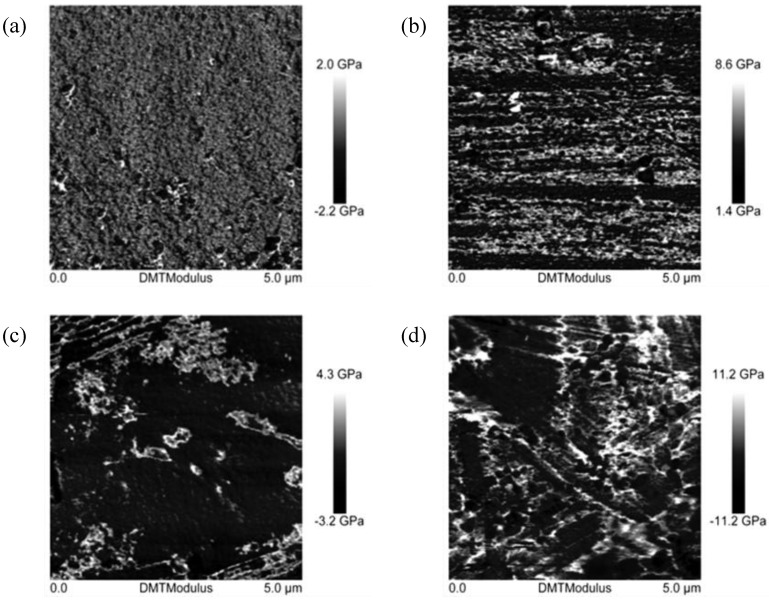
AFM images obtained by QNM for the surface of samples of unfilled EP (**a**) and for composites: (**b**) EPBP1-3.0; (**c**) EPIHSP3-3.0; (**d**) EPBP1IHSP3-3.0.

The AFM microscope images (Figure 17) showing the distribution of Young’s modulus for the surface of the composite EPBP1-3.0 containing 3.0% BP1 (Figure 17b) show a uniform dispersion of nanofiller in the form of bright areas—a high Young’s modulus of the polymer matrix can be observed. The average nanofiller particle size determined on the basis of the size of these areas is 52.2 nm. Similar areas were not observed for the unfilled EP (Figure 17a). In the case of the composites EPIHSP3-3.0, with the same content of modified silica nanofiller, small agglomerates are visible (Figure 17c), which in the composite EPBP1IHSP3-3, containing 1.5% BP1 and 1.5% I-HS-P3 (Figure 17d) are reduced, thereby lowering the average particle size from 76.6 to 60.5 nm. Differences in the topography of the nanocomposites, in terms of the calculated average roughness (Ra), were also observed. The average roughness increased from 3.57 nm for the unfilled epoxy resin to 5.53 nm for EPBP1-3.0, to 6.2 nm for EPIHSP3-3.0, and to 10 nm for EPBP1IHSP3-3.0. To eliminate noise before determining the roughness, a first-order smoothing function was used.

### 3.9. Effect of Nanofillers on the Flammability of Composites

Measurements of oxygen index (LOI) showed that the addition of the nanofillers BP1, BP2 and POSS-modified silica (I-HS-P3) to the polymer matrix led to higher values of the index (Table 6). The highest increase in the oxygen index was observed for the composites EPBP1-3.0, EPBP1IHSP3-3.0 and EPBP2IHSP3-3.0, for which the LOI was respectively 28.1%, 29.2% and 27.6%, compared with 19.0% for the unfilled resin (EP).

Tests of the UL94 flame resistance of samples of the composites confirmed the conclusions of the LOI results (Table 6). The best flame resistance, class V1, was obtained for composites with 3% content of the filler BP1 and the hybrid fillers BP2IHSP3 and BP1IHSP3. Linking these results with the previously discussed structural analysis helps demonstrate that the composites with these filler contents are nanocomposites with exfoliated structure. This design makes the best use of the reinforcing and flame retardant properties of these fillers. Singly modified silica (HS-I-P3) does not work as efficiently, as the composite samples containing it reached only HB flammability class.

**Figure 18:**
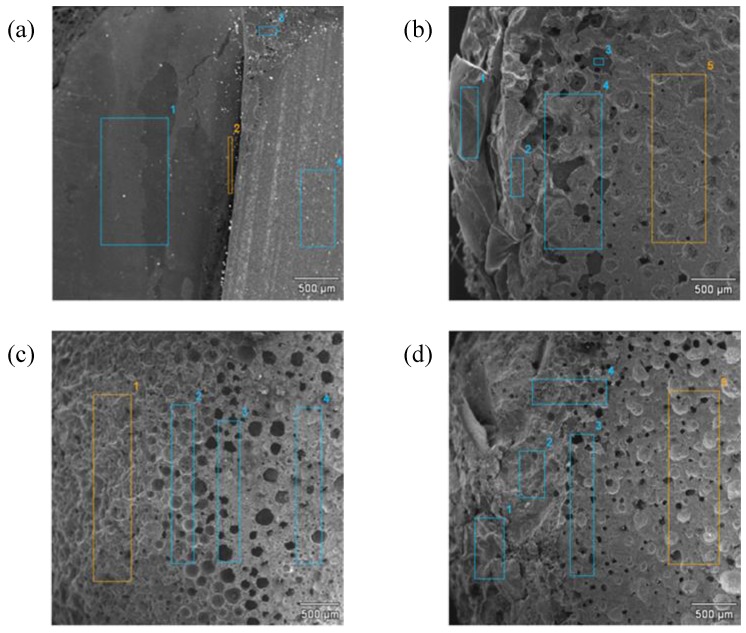
SEM microphotographs and EDS analysis results of samples of burnt EP (**a**) and composites; (**b**) EPBP1-3.0; (**c**) EPIHSP3-3.0; (**d**) EPBP1IHSP3-3.0. Numbers 1,2,3,4,5, denoted places of EDS analysis of samples sections (see Table 8).

SEM analysis of the burnt samples of EP and the composites EPBP1-3.0, EPIHSP3-3.0, and EPBP1IHSP3-3.0, together with elemental analysis using the EDS technique, showed that in the case of composites with the addition of 3% BP1, porous sinter is formed in the burnt part (see Figure 18b). There is also increased silicon content in the burnt part (Table 8), which may indicate the possible formation of a glassy layer of silicon carbide. Such a glassy-porous structure improves the flame retardant properties of the composite, as it impedes the access of oxygen necessary to sustain the flame, and inhibits the outflow of exhaust gases acting as retardants. The visible highly porous layer provides thermal insulation over the burning composite. In the case of composites containing 3% modified silica (EPIHSP3-3.0), a more developed porous structure is formed (see Figure 18c). Higher Si concentration can be observed in the burnt part, which may result from the formation of silicon carbide.

In the burnt part of composite sample EPBP1IHSP3-3.0, containing both nanofillers, a glassy-porous structure can be seen (Figure 18d), as in the case of the composite with modified bentonite shown in Figure 18b. The results of EDS microanalysis of chemical composition confirmed the previous observations on Si agglomeration in the burnt part of composite sample EPBP1IHSP3-3.0.

**Table 8:** Results of EDS analysis of surface composition of burnt samples of EP and the composites EPBP1-3.0, EPIHSP3-3.0, EPBP1IHSP3-3.0.

Symbol of composite	Area number	Content of elements, %				
		C	N	O	Si	Cl
EP	1	92.45	3.44	3.47	0.56	0.08
	2	91.51	3.36	4.60	0.49	0.04
	3	91.17	3.21	5.20	0.38	0.04
	4	88.43	3.86	7.56	0.06	0.09
EPBP1-3.0	1	86.14	8.22	3.69	1.85	0.01
	2	88.09	6.27	3.79	1.84	0.01
	3	87.40	6.21	5.00	1.37	0.02
	4	88.80	5.65	3.92	1.61	0.02
	5	89.78	5.74	3.26	1.18	0.04
EPIHSP3-3.0	1	85.59	5.81	6.74	1.84	0.02
	2	87.25	5.33	5.64	1.76	0.02
	3	86.10	5.79	6.35	1.73	0.03
	4	86.66	5.52	6.14	1.64	0.04
EPBP1IHSP3-3.0	1	85.97	8.22	3.69	2.11	0.01
	2	87.89	6.27	3.79	2.04	0.01
	3	86.90	6.21	5.00	1.87	0.02
	4	88.40	5.65	3.92	2.01	0.02
	5	89.18	5.74	3.26	1.78	0.04

## 4. Conclusions

New hybrid nanofillers, based on bentonite and silica modified with polyhedral oligomeric silsesquioxane, have been produced. The functional hybrid nanofillers exhibit good miscibility with the epoxy resin, with no sedimentation of the composite. The addition of hybrid nanofillers slightly prolongs the gelation time of the studied composites. The addition of 1.5–4.5 wt.% of the fillers BP1 and I-HS-P3 to EP produced a marked improvement in the strength properties of the composites: a significant unnotched impact strength and ultimate tensile strength and a significant increase in Young’s modulus.

The introduction of the studied fillers to EP significantly improved the flammability limit of the composites. The best results were obtained for the hybrid composites with a total filler content of 3.0%. The efficient dispersal of the hybrid nanofillers at a nanometric level in the matrix was confirmed by WAXS, SEM, and TEM techniques. The formation of an exfoliated structure was also confirmed. SEM analysis coupled with EDS confirmed that the applied hybrid nanofillers act via the mechanism of the flame retardancy of the composite in the solid phase [7].
